# Performance and Dimensionality of Pretreatment MRI Radiomics in Rectal Carcinoma Chemoradiotherapy Prediction

**DOI:** 10.3390/jcm13020421

**Published:** 2024-01-12

**Authors:** Mladen Marinkovic, Suzana Stojanovic-Rundic, Aleksandra Stanojevic, Aleksandar Tomasevic, Radmila Jankovic, Jerome Zoidakis, Sergi Castellví-Bel, Remond J. A. Fijneman, Milena Cavic, Marko Radulovic

**Affiliations:** 1 Clinic for Radiation Oncology and Diagnostics, Department of Radiation Oncology, Institute for Oncology and Radiology of Serbia, 11000 Belgrade, Serbia; mladen309@gmail.com (M.M.); stojanovics@ncrc.ac.rs (S.S.-R.); aleksandar.tomasevic@ncrc.ac.rs (A.T.); 2 Faculty of Medicine, University of Belgrade, 11000 Belgrade, Serbia; 3 Department of Experimental Oncology, Institute for Oncology and Radiology of Serbia, 11000 Belgrade, Serbia; astefanovic496@gmail.com (A.S.); jankovicr@ncrc.ac.rs (R.J.); milena.cavic@ncrc.ac.rs (M.C.); 4 Department of Biotechnology, Biomedical Research Foundation, Academy of Athens, 11527 Athens, Greece; izoidakis@bioacademy.gr; 5 Department of Biology, National and Kapodistrian University of Athens, 15701 Athens, Greece; 6 Gastroenterology Deparment, Fundació de Recerca Clínic Barcelona-Institut d’Investigacions Biomèdiques August Pi i Sunyer, Centro de Investigación Biomédica en Red de Enfermedades Hepáticas y Digestivas, Clínic Barcelona, University of Barcelona, 08036 Barcelona, Spain; sbel@recerca.clinic.cat; 7 Department of Pathology, The Netherlands Cancer Institute, 1066 CX Amsterdam, The Netherlands; r.fijneman@nki.nl

**Keywords:** rectal carcinoma, radiomics, neoadjuvant, chemoradiotherapy, MRI

## Abstract

(1) Background: This study aimed to develop a machine learning model based on radiomics of pretreatment magnetic resonance imaging (MRI) 3D T2W contrast sequence scans combined with clinical parameters (CP) to predict neoadjuvant chemoradiotherapy (nCRT) response in patients with locally advanced rectal carcinoma (LARC). The study also assessed the impact of radiomics dimensionality on predictive performance. (2) Methods: Seventy-five patients were prospectively enrolled with clinicopathologically confirmed LARC and nCRT before surgery. Tumor properties were assessed by calculating 2141 radiomics features. Least absolute shrinkage selection operator (LASSO) and multivariate regression were used for feature selection. (3) Results: Two predictive models were constructed, one starting from 72 CP and 107 radiomics features, and the other from 72 CP and 1862 radiomics features. The models revealed moderately advantageous impact of increased dimensionality, with their predictive respective AUCs of 0.86 and 0.90 in the entire cohort and 0.84 within validation folds. Both models outperformed the CP-only model (AUC = 0.80) which served as the benchmark for predictive performance without radiomics. (4) Conclusions: Predictive models developed in this study combining pretreatment MRI radiomics and clinicopathological features may potentially provide a routine clinical predictor of chemoradiotherapy responders, enabling clinicians to personalize treatment strategies for rectal carcinoma.

## 1. Introduction

Colorectal carcinoma was the third most common cancer and the second most fatal cancer with an estimated 1.4 million new cases worldwide in 2020 [1]. Due to the increasing incidence in younger people [2,3], the American Cancer Society now recommends screening starting at age 45 [4].

Rectal carcinoma prognosis varies by stage, with 5-year survival rates of over 90% for early-stage and ~20% for late-stage disease [5]. The standard treatment for locally advanced rectal carcinoma is neoadjuvant chemoradiotherapy (nCRT) followed by total mesorectal excision, with or without adjuvant chemotherapy [6]. This treatment is highly effective, with low rates of local recurrence [6].

The clinical assessment of neoadjuvant treatment response for rectal carcinoma occurs before surgery, using MRI scans. Clinical complete response (cCR) is determined as the absence of residual disease in the rectum after neoadjuvant treatment, as confirmed by digital rectal examination, endoscopic evaluation (rectosigmoidoscopy), and control MRI examination. Another method of assessing treatment response is tumor regression grading (TRG), which is based on the histopathological examination of tumor tissue postoperative specimen. TRG is graded on a scale from 1 to 5, with TRG1 representing the best response and TRG5 indicating the poorest response, characterized by no observable tumor regression. TRG1 corresponds to pathohistological complete regression (pCR), which is observed in 10–30% of cases [7] and defined as the absence of viable tumor cells after total mesorectal excision (ypT0N0). It has been shown that pCR is associated with improved outcomes, regardless of the initial clinical T and N stages of the disease [8].

TRG is an important prognostic factor for local recurrence, disease-free survival, and overall survival (OS) of rectal carcinoma patients. Patients with TRG 1-2 (good responders) have significantly better outcomes than poor responders with TRG 3-5 [9].

More reliable predictors of nCRT response are needed to enable precision medicine in routine clinical practice, thereby maximizing the effectiveness of existing treatments. Personalized treatment for chemoradioresistant patients may include second-line chemotherapy, participation in experimental trials with more targeted therapies, more intense radiotherapy, or combination therapy with immunotherapy. Conversely, to improve the quality of life for patients responding well to neoadjuvant treatment, adjustments such as less invasive surgery or a non-operative approach (“watch and wait”) may be considered. However, such precision in clinical decision making is hindered by the limited reliability of current clinical, pathological, and molecular predictors of chemoradioresistance in rectal carcinoma, such as tumor stage, tumor regression grade, tumor markers (carcinoembryonic antigen), circulating tumor DNA, DNA methylation level, and cancer-related inflammatory markers [10,11].

In our previous work, we compared the proteomic profiles of LARC patients who responded well and poorly to nCRT [12]. We also identified methylenetetrahydrofolate reductase (MTHFR) 667C and 1298A alleles as low-penetrant risk factors for rectal cancer [13]. Moreover, we investigated the predictive value of clinicopathological features in rectal carcinoma [14]. In this study, we enhance our predictive research by integrating radiomics analysis.

Radiomics analysis of magnetic resonance imaging (MRI) scans can provide valuable information for predicting nCRT response in rectal carcinoma, complementary to traditional clinical and molecular methods. Radiomics extracts quantitative data from medical images that are not visible to the naked eye. Most predictive imaging models for rectal carcinoma previously used PET scans [15,16], while several used MRI. Some of these studies have used post-nCRT MRI to assess treatment response rather than predict it [17,18,19]. Other studies have used pretreatment MRI to predict nCRT response, predominantly relying on features computed from unfiltered images [20], with occasional inclusion of wavelet [21] and Laplacian of Gaussian (LOG) filters [22,23,24]. Image filters are used in MRI radiomics to better emphasize texture or structural information. However, due to the limited use of filters in radiomics analysis of rectal carcinoma, the predictive potential of several commonly used filters, such as logarithmic, exponential, square, square root, exponential and LBP, has remained unexplored. Additionally, the limited use of image filters in previous predictive radiomics MRI studies has prevented the direct evaluation of how radiomics dimensionality affects predictive performance. Notably, a recent study investigating the effect of image filtering on radiomics features recommended conducting radiomics analysis using all available filters [25].

Consequently, to address the clinical need for improved prediction of nCRT response in rectal carcinoma, this retrospective study systematically evaluated the predictive potential of all available image filters and dimensionality in radiomics MRI analysis.

## 2. Materials and Methods

We utilized a retrospective cohort of patients with locally advanced rectal carcinoma (LARC) who have undergone diagnostic MRI and neoadjuvant chemoradiotherapy. Pretreatment MRI scans were subjected to radiomics feature extraction, encompassing morphological and textural aspects of the tumor. The least absolute shrinkage selection operator (LASSO) was then employed to train and validate the predictive model, employing statistical and cross-validation techniques to select the predictively most valuable features, thus ensuring robustness and generalizability.

### 2.1. Ethics Approval Statement

The study received approval from the Ethics Committee of the Institute for Oncology and Radiology of Serbia (Approval No. 2211-01 from 11 June 2020) and Ethics Committee of the Faculty of Medicine, University of Belgrade (Approval No. 1322/XII-17 from 3 December 2020). It adheres to The Code of Ethics of the World Medical Association (Declaration of Helsinki), as published in the British Medical Journal (18 July 1964) and its 7th revised edition in 2013. All patients signed an informed consent.

### 2.2. Patients

A total of 75 patients diagnosed with LARC were enrolled in this study. The patient cohort was selected from patients treated at the Institute for Oncology and Radiology of Serbia, spanning the period between June 2020 and January 2022. Inclusion criteria required patients to have histopatologically confirmed adenocarcinoma of the rectum, with the tumor located within up to 12 cm from the anal verge, as determined by rigid proctoscopy. LARC was defined as encompassing T3-T4N0 stages or any T stage with positive lymph nodes (N+). Pretreatment assessment included abdominal and pelvic MRI scans, as well as computed tomography (CT) scans or chest X-rays. All patients underwent long-course nCRT. Radiotherapy (RT) was administered using the volumetric modulated arc therapy-simultaneous integrated boost technique (VMAT-SIB). The prescribed dose to the mesorectum and pelvic lymph nodes was 45 Gy, administered in daily fractions of 1.8 Gy. Additionally, a simultaneous integrated boost (SIB) was administered to the macroscopic disease region with a 2 cm margin, totaling 54 Gy, delivered in daily fractions of 2.16 Gy. Concomitant chemotherapy was initiated on the first day of radiotherapy and continued during the first and fifth weeks of the treatment regimen. The chemotherapy regimen comprised 5-fluorouracil (5-FU) at a dose of 350 mg/m^2^ on the first day of the first and fifth weeks of radiotherapy, along with leucovorin (25 mg/m^2^ daily) administered during the five consecutive days of the first and fifth weeks of radiotherapy.

**Figure 1:**
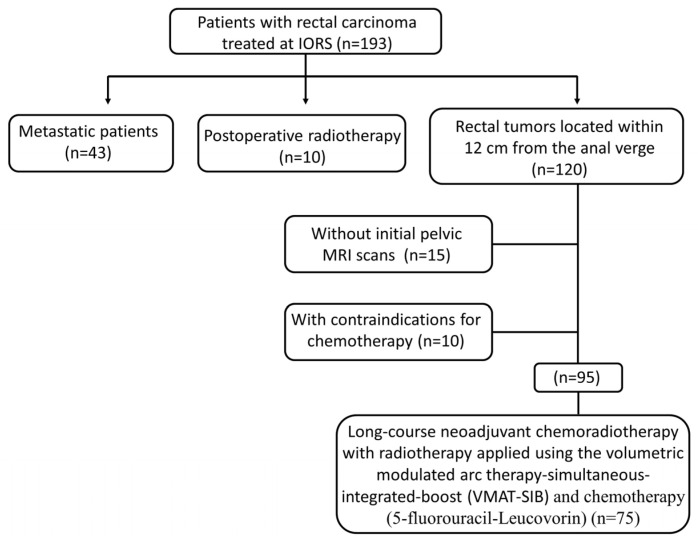
Flow chart of patient selection.

The evaluation of tumor response was conducted eight weeks following the completion of nCRT and included pelvic MRI scans, rigid proctoscopy, and digital rectal examination. For patients achieving complete clinical response (cCR) and initially distant tumor location, immediate radical surgery was not recommended. Instead, they were enrolled in a stringent follow-up program (“watch and wait” approach). Patients with cCR who were candidates for sphincter preservation surgery underwent surgical resection within a window of eight to twelve weeks following the completion of nCRT. For patients exhibiting a partial response (PR), surgery was conducted approximately twelve to fifteen weeks after the completion of nCRT. Patient selection overview is shown in Figure 1.

After the completion of neoadjuvant chemoradiotherapy (nCRT) among 63 patients who underwent operative treatment, 37 (59%) had surgery at the Clinic for Digestive Surgery, University Clinical Center, Belgrade, while 26 patients (41%) underwent surgery at the Institute for Oncology and Radiology, Belgrade.

Patients’ responses to treatment were categorized using the Mandard classification system based on the pathohistological TRG observed in postoperative specimens. Responders included patients with cCR who did not require surgery, as well as those with TRG1 and TRG2 postoperative categories. Non-responders encompassed patients classified as TRG 3–4.

### 2.3. MRI

Initial MRI data of the pelvic region were available for 71 out of the total 75 patients included in this study. All MRI examinations featured 1.5-Tesla 3D T2-weighted (T2W) contrast sequences, which were utilized for precise tumor delineation. MRI were acquired at eight institutions, using the following scanner models: Siemens Magnetom Avanto Fit 1.5 T, Siemens Magnetom Symphony TIM 1.5 T, and Hitachi Echelon 1.5 T. To minimize the effect of using different scanners, voxel intensities within tumor volumes of interest were normalized by the z-score method prior to radiomics analysis.

Intra-observer reproducibility was assessed by comparing two segmentations performed by the same observer (M.M.), 8 weeks apart to reduce recall bias. The similarity between segmentations was estimated using the dice similarity coefficient (DSC), calculated with a 3D Slicer (version 5.4.0). The mean (±standard deviation) intra-observer DSC was 0.936 ± 0.029 (range: 0.872–0.970). The mean (±standard deviation) intra-observer average Hausdorff distance was 0.50 ± 0.04 mm (range: 0.11–1.39).

### 2.4. Postprocessing

The initial step in our analysis involved the import of MRI Digital Imaging and Communications in Medicine (DICOM) files into the 3D Slicer and the subsequent generation of multiple resolution bitmap (MRB files), streamlining the data for further examination [26]. For all 71 patients under consideration, a precise segmentation of rectal carcinoma tumor volumes, which refers to the process of delineating regions of interest (ROI), was carried out. Sequences exhibiting any artifacts were excluded from the analysis to ensure the highest possible image quality.

**Figure 2:**
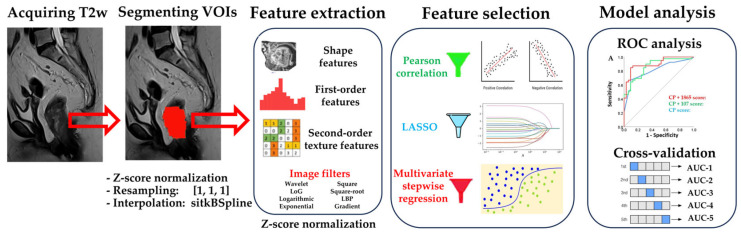
Radiomics analysis workflow. Prior to radiomics analysis, images were z-score normalized, resampled, and interpolated. The primary rectal tumors were segmented on T2-weighted MRI images acquired before treatment. Sagittal MRI T2W sequence is shown with a red area corresponding to the segmented primary rectal tumor. All available image filters were applied to the images and all feature classes were computed. Feature values were also z-score normalized. We performed feature preselection by discarding features without a significant association with the outcome. LASSO and multivariate stepwise linear regression were used for feature selection to create two models: one medium-dimensional and one very high-dimensional.

Tumor volume delineation was a collaborative effort involving both a radiation oncologist and reference to the initial radiologist reports, which provided critical guidance in ensuring accuracy and consistency. The radiation oncologist (M.M.) with six years of expertise in oncologic imaging manually segmented the volume of interest (VOI) on all 71 T2-weighted images using the open-source 3D Slicer software, v5.4.0. The segmentation of the tumor was performed on each image slice where the tumor was visible. Figure 2 shows the MRI sagittal plane before and after segmentation.

### 2.5. Sample Size Calculation

The prospective sample size calculation was based on a pilot experiment involving 36 patients. It required 40 patients with 18 positive cases for alpha = 0.05, beta = 0.20, and area under the ROC curve AUC = 0.75 (Medcalc 14.8.1; MedCalc Software Ltd., Ostend, Belgium). The actually obtained AUCs for the two calculated scores including clinicopathological and radiomics features were 0.86 and 0.90, with a final sample size of 71 patients.

### 2.6. MRI Normalization

Z-score normalization was applied to MRI images as instructed by the parameter file listed below. Prior to radiomics feature extraction in 3D, all images and segmentations were resampled and interpolated to obtain isotropic voxels of 1 mm^3^.

### 2.7. Feature Extraction

The computational analysis of rectal MRI images was conducted using the open-source Pyradiomics plugin, integrated into 3D Slicer, named “Radiomics” [27]. PyRadiomics is compliant with Imaging Biomarker Standardization Initiative (IBSI) standards. The Pyradiomics software was customized through the parameter file provided below, instructing the generation of all available image transformations and feature types, resulting in the computation of a total of 2141 features per image.
setting:  normalize: true  normalizeScale: 100  resampledPixelSpacing: [1, 1, 1]  interpolator: ’sitkBSpline’ 
imageType:  Original:     binWidth: 10.0  LoG:     sigma: [1.0, 2.0, 3.0, 4.0, 5.0]    binWidth: 3.0  Wavelet:     binWidth: 3.0  Square:     binWidth: 4.0  SquareRoot:     binWidth: 8.0  Logarithm:    binWidth: 9.0  Exponential:    binWidth: 3.0  Gradient:    binWidth: 3.0  LBP2D:     binWidth: 0.1  LBP3D:     binWidth: 0.2
featureClass:  glcm:  firstorder:  shape2D:  shape:  glrlm:  glszm:  gldm:  ngtdm:

The width of discretization bins for feature extraction was adjusted separately for each image filter in a pilot analysis of 37 MRI, targeting a bincount range between 30 and 130. Feature extraction was performed on the original images (107 features) as well as after applying eight types of built-in preprocessing filters: wavelet (5 × 93 = 465 features), square (93 features), square root (93 features), logarithm (93 features), gradient (93 features), exponential (93 features), Laplacian of Gaussian (LoG, 5 × 93 = 465 features), local binary pattern (LBP 2D, 89 features; LBP 3D 3 × 92 = 276). Wavelet filtering yields 8 subbands, all possible combinations of applying a high or a low pass filter in each of the three dimensions.

Among the basic set of 107 original features, 18 were first-order intensity and 14 shape, while the remaining 75 were second-order texture features belonging to six distinct classes: gray-level co-occurrence matrix (GLCM, 24 features), gray-level run length matrix (GLRLM, 16 features), gray-level size zone matrix (GLSZM, 16 features), gray-level dependence matrix (GLDM, 14 features), and neighboring gray-tone difference matrix (NGTDM, 5 features). We adhered to the common assumption of dimensionality: moderate: 10–100 dimensions, high dimensionality: 100–1000 dimensions, and very high dimensionality: 1000+ dimensions. Detailed descriptions of the extracted radiomics features can be found in the PyRadiomics documentation available at https://pyradiomics.readthedocs.io/en/latest/features.html, accessed on 26 October 2023.

### 2.8. Normalization of the Calculated Feature Values

The computed variable values ranged between 6.24 × 10^10^ and −3.81 × 10^6^. By employing z-score normalization, we were able to bring values of all variables to a similar scale, with values ranging between −6.60 and 8.31, thus enabling better comparison among features.

### 2.9. Evaluation of Predictive Performance

Evaluation of the predictive performance for the demographic, MRI, clinicopathological, and the radiomics features was approached by the univariate linear regression and receiver operating characteristic (ROC) analysis, both executed using the IBM SPSS software package version 28 by the IBM Corporation in Armonk, NY, United States. Our study focused on the prediction of the response to chemoradiotherapy in rectal carcinoma as the primary endpoint. In the case of univariate linear regression, we worked with continuous values for independent variables, while the dependent endpoint was also expressed in continuous numerical values, ranging from 0 to 4. Specifically, we assigned the values as follows: cCR = 0, TRG1 = 1, TRG2 = 2, TRG3 = 3, and TRG4 = 4. Linear regression assesses whether independent predictor variables explain the dependent variable (endpoint or outcome).

ROC analysis requires a categorized dependent endpoint variable. The area under the rate of change curve (AUC) was used to evaluate the discriminatory efficiency of the prognostic features in relation to a binary endpoint. Discrimination refers to the ability of the prognostic features to classify patients based on their actual metastasis occurrence. AUC values range from 0.0 to 1.0, where 0.5 indicates random chance discrimination, while values of 0.0 or 1.0 indicate perfect discrimination. Furthermore, it is of note that the AUC not only quantifies the level of discrimination, but also offers directional information. For instance, AUC values of 0.2 and 0.8 suggest the same level of discriminatory efficiency but in opposite directions. We set a significance threshold at *p* ≤ 0.05 to determine the statistical significance of our findings.

### 2.10. Model Selection

Predictive models were constructed using the features selected by LASSO regression (Stata/MP 17, StataCorp, College Station, TX, USA) followed by stepwise multivariate linear regression (IBM SPSS v28, IBM Corporation, Armonk, NY, USA). LASSO was performed by the inclusion of radiomics features together with all 72 clinicopathological features.

Although LASSO handles multicollinearity and removes features unrelated to the outcome, we performed feature preselection, prior to LASSO and multivariate regression. Radiomics features which did not significantly correlate with the outcome by Pearson coefficient were discarded. Dimensionality was further reduced by the use of LASSO regression (Stata/MP 17, StataCorp, College Station, TX, USA), followed by backwards stepwise multivariate binary regression (IBM SPSS v28). Clinicopathological features were exempt from LASSO feature selection because only five features were significantly associated with the outcome. LASSO regression is a machine learning regularization method that selects covariates and estimates their coefficients using a tuning parameter λ in cross validation. As λ increases, LASSO eliminates less important variables by shrinking their coefficients to zero, providing robustness against outliers and improving generalization performance. Multivariate stepwise backwards linear regression proceeds to remove the least significant variables, one at a time. The removal of variables continues until the *p* value of the next feature to be removed falls below 0.05, satisfying the stopping rule.

Features remaining after the applied selection methods were used for computation of the radiomics scores, based on the selected features and the coefficients provided by the regression methods, using the formula: variable1×coefficient1 + variable2 × coefficient2 + variable3 × coefficient3 + ….

### 2.11. Validation

To mitigate the over-optimistic bias in the univariate and multivariate logistic regression analysis, the bootstrap technique was employed, generating 5000 random resamples of the data, allowing for more robust estimation [28]. The original confidence intervals (95% CIs) and *p* values were adjusted using this resampling approach, ensuring more reliable results (IBM SPSS v28).

In addition, split-sample cross validation was utilized as a validation method to determine the optimal penalty coefficient λ in the LASSO regression analysis conducted in Stata/MP 17. This technique involved dividing the data into two subsets: a training set and a validation set. The training set was used to fit the LASSO regression model and select the most informative features, while the validation set was used to evaluate the performance of the model.

## 3. Results

The predictive model comprised two scores: a moderately dimensional score derived from 72 clinicopathological (CP) and 107 radiomics features, and a very highly dimensional score derived from 72 CP and 1862 radiomics features. We also computed a CP score to serve as the benchmark for state-of-the-art performance in predicting nCRT response. The nCRT continuous outcome was defined in the order: clinical complete response (cCR), tumor regression grade 1 (TRG1), TRG2, TRG3, and TRG4. For receiver operating characteristic (ROC) analysis, we categorized this outcome into responders (cCR, TRG1, TRG2) and non-responders (TRG 3–4) [21].

Table 1 provides a comprehensive overview of patient characteristics, disease specifics, treatment details, and outcomes. Notably, direct tumor spread was the primary form of involvement when the mesorectal fascia was affected. Pathological regional lymph nodes were present in 97.3% of cases, and one-third of patients displayed extramural vascular invasion (EMVI). In our cohort, 46.7% of patients were categorized as treatment responders.

We observed that LASSO sometimes selected features that were weakly associated with the outcome. Therefore, we removed all features that did not significantly correlate with the outcome before applying LASSO feature selection. This preselection improved the predictive performance of the scores selected by LASSO. In contrast, removing highly correlated features before LASSO did not affect the final results. The workflow of our analysis is presented in Figure 2.

Table 2 presents features achieving the strongest predictive performance, from each of the three categories: CP features, radiomics features computed from original MRI images, and radiomics features computed from both original and filtered MRI images. Notably, out of the 72 available CP features, only the five presented here are significantly associated with the outcome. Univariate predictive analysis of the features in Table 2 using the Pearson correlation test and univariate linear regression showed that individual features from the CP and two radiomics feature groups had comparable associations with the outcome.

We calculated a basic set of 107 radiomics features from the original images, including 14 size/shape, 18 intensity, and 75 second-order texture features. To enhance the depth of our analysis, we applied 21 image transformations using various filters, including LoG with five settings, wavelet with eight subbands, LBP 3D with two settings, and square, square root, logarithmic, exponential, and gradient filters. This increased the total number of features to 2141. However, we removed 279 features computed on images produced by wavelet decomposition into LHH, HLH, and HHH subbands due to the low gray level intensity ranges. This reduced the total number of features to 1862.

To address the high dimensionality of the data and improve the predictive performance of the final models, we preselected radiomics features by removing those that did not show a significant Pearson correlation with the outcome. This reduced the number of radiomics features from 1862 to 365. We did not preselect CP features because only five of them were significantly associated with the outcome.

After preselection, we used linear LASSO regression followed by multivariate stepwise backward linear regression to select features. The aim of feature selection is to eliminate redundant features and those that are irrelevant to the outcome. From the initial two radiomics sets comprising 72 CP + 107 radiomics features and 72 CP + 1862 radiomics features, LASSO selected nine and eleven features, respectively. Multivariate linear regression further refined the models to five features each (Table 3).

**Figure 3:**
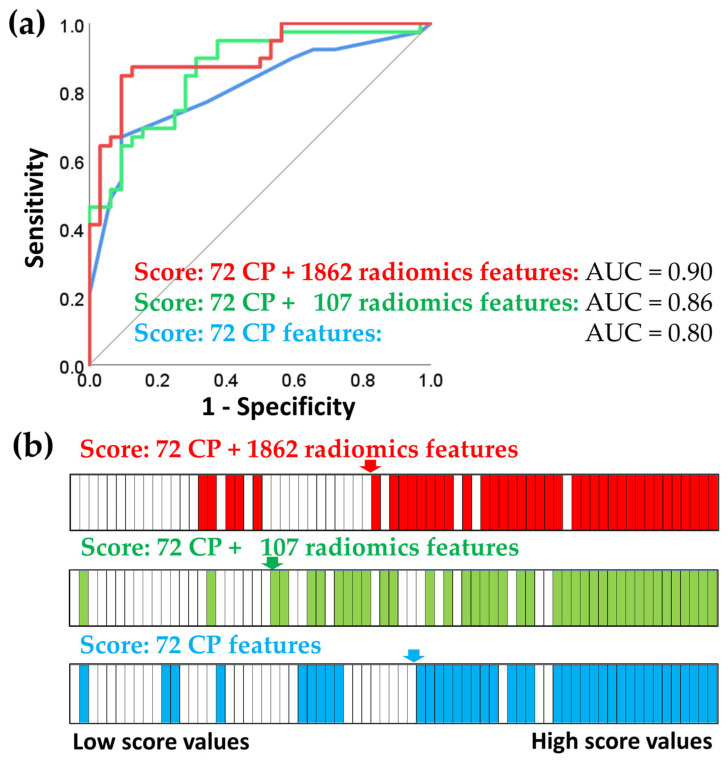
ROC analysis of three prognostic scores for chemoradiotherapy response. The chemoradiotherapy response was stratified into two categories: responder (cCR, TRG1, and TRG2) and non-responder (TRG3, TRG4). (**a**) ROC curves for the predictive scores: 72 clinicopathological features (CP) score, 72 CP + 107 radiomics features score, and 72 CP + 1862 radiomics features score. (**b**) Simplified visual representation of the classification between responders (white) and non-responders (blue, green or red). The continuous values of the prognostic scores are ordered from lowest (left) to highest (right). This figure illustrates that as the score values increase, the likelihood of being a non-responder patient also increases.

To assess the benefits of comprehensive and very high-dimensional tumor characterization, we compared the predictive performance of the two models described above: one derived from a smaller set of 107 features and one derived from a much larger set of 1862 radiomics features (Table 3). Table 3 shows that the very high-dimensional model had only a marginal improvement in predictive capability, with an R-squared value of 0.47, compared to the R-squared value of 0.45 for the model with 107 features. This finding was consistent with the ROC analysis presented in Figure 3.

ROC analysis requires a binary outcome; therefore, we categorized patients into responders (cCR, TRG1, and TRG2) and non-responders (TRG3 and TRG4) (Figure 3). cCR means that no cancer was detected in the primary tumor by imaging and physical examination. TRG ranges from TRG1 and TRG2 (complete response and near complete response, respectively) to TRG3 and TRG4 for moderate and poor response. Figure 3 shows that the score with the highest dimensionality computed using 72 CP and all 1862 radiomics features had the best predictive performance.

Table 4 presents the five-fold stratified cross validation to evaluate the generalizability of the top-performing predictive model presented in Table 3 and Figure 3. The cohort was split into five evenly sized training and validation folds (subsets of the dataset). Importantly, to avoid data leakage, validation data were not used during training. It is of note that the model selected from 72 CP + 1862 radiomics features using the entire patient cohort (Table 3) achieved very a similar predictive performance (AUC = 0.90) as the five models derived from the same pool of features but were constructed separately in each of the randomly split training folds (average AUC = 0.91; Table 4). When these models were tested on matching unseen validation folds, the predictive performance decreased to an average of 0.84 (Table 4).

**Figure 4:**
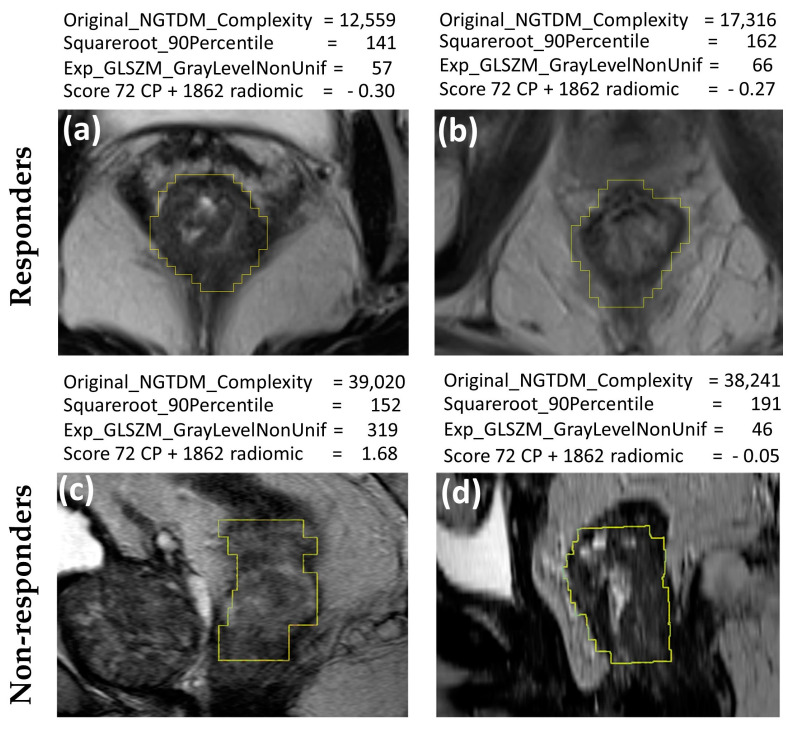
Examples of analyzed MRI images in the T2W contrast sequence sagittal plane (**a**,**b**) nCRT responders with cCR and (**c**,**d**) nCRT non-responders with TRG4. The values of several predictively prominent features selected for the predictive scores (Table 3) are indicated for each image. Notably, these feature values are in their native version, except for the score 72 CP + 1862 radiomics values, which have been z-score normalized.

Representative MRI images of nCRT responders and non-responders are shown in Figure 4. The computed values of features (Figure 4) illustrate the classification of images based on nCRT response. In the case of these four patients, original NGTDM complexity and 72 CP + 1862 radiomics scores were consistently higher in non-responder patients, while the square root 90th percentile and exponential gray level nonuniformity were not consistent. This association of increasing feature values with increasing TRG (reflecting lower response to nCRT) was in line with the data presented in Table 1, because these features yielded AUC values above 0.5. The texture features of tumors are subvisual, making it difficult to visually distinguish between images of tumors from responder and non-responder patients (Figure 4).

## 4. Discussion

The standard of care in LARC is nCRT followed by radical surgery. However, patient responses to nCRT vary widely, and there is a lack of effective methods to identify patients who would or would not benefit from the treatment. In this study, we developed two novel MRI-based radiomics models to predict the response of LARC to nCRT. We also examined the relationship between the depth of radiomics analysis and predictive performance. Without an external cohort to evaluate the generalizability of our models, we showed that the predictive performance was mostly retained in unseen validation folds using cross validation.

This study highlights the complementary predictive power of clinicopathological and radiomics features. The association of mucinous tumor differentiation with poor treatment response was consistent with the findings of Simha et al. [29]. Similarly, MRI-derived EMVI was associated with poor responses in this study, as in previous findings [30]. This EMVI-associated chemoradioresistance may be explained by tumor hypoxia, prompting the investigation of dose escalation with adaptive MRI-guided radiotherapy for this group of patients [31]. The identification of basophil counts as a predictive parameter in LARC was also consistent with our previous study [14] and findings in advanced gastric cancer [32].

In line with most previous predictive studies in rectal carcinoma that used radiomics analysis of MRI data, we investigated the T2W sequence. This sequence is considered the most suitable for predictive purposes because it can capture predictive clues well, is less susceptible to artifacts, and has good reproducibility [33].

With the inclusion of all available image filters, this study reports that the features selected for predictive scores are derived from the original images and those generated using previously unutilized exponential and square root filters. This finding is consistent with a previous study that investigated the impact of preprocessing filters on the predictive performance of radiomics analyses across seven radiomics datasets [34], whereby square root, exponential, and wavelet filters achieved the best predictive performance [34]. The exponential filter can enhance image boundaries and contrast, while the square root filter highlights subtle textural patterns that might otherwise be difficult to detect and has noise reduction properties.

Omics methodologies like radiomics exploit the advantages of comprehensive approaches involving thousands of features. However, using many features risks false discoveries, where statistical significance may arise by chance. One goal of this study was to assess the predictive benefits of the deepest radiomics approach. To achieve this, we compared predictive models based on large and small numbers of radiomics features. The model trained on 1862 features had a moderate predictive advantage over the model trained on 107 features (AUC 0.90 vs. 0.86). This suggests that using a very large number of features can capture additional predictively relevant information, even if a moderately dimensional analysis with only 107 features already delivers very good predictive performance. Although the improvement from AUC 0.86 to 0.90 may seem incremental, further improvements become increasingly difficult as predictive performance nears the ideal AUC of 1.0. Therefore, even small improvements in predictive performance by 0.04 AUC can have a substantial clinical value, reducing the number of incorrectly classified patients by 5%.

In this study, we developed radiomics signatures with three to five features after dimensionality reduction, while previous studies used up to 30 features [18,19,35]. A common guideline is to use one feature for every ten patients [36,37]. As expected, our predictive scores, which integrated both clinicopathological and radiomics features, outperformed the benchmark predictive score based only on clinicopathological features. This finding aligns with the previous report that radiomics features add value when combined with qualitative MRI features [20] while most studies did not provide such comparison. However, some studies have reported clinicopathological scores outperforming models that incorporate radiomics features [38].

The fact that the MRI data were acquired at eight different institutions, presented an advantage for this study by increasing its generalizability. Importantly, issues related to comparability arising from diversity in MRI acquisition protocols and scanner models were addressed through the standardization of voxel intensities using z-score normalization. While chemoradiotherapy responsiveness is a surrogate endpoint that provides valuable insights into treatment efficacy and short-term outcomes, it is important to recognize its limitations because it serves as a measure for the true clinical outcomes of interest, like disease recurrence or long-term survival [39]. Not all patients who respond to nCRT will experience long-term disease control and conversely, not all non-responders will have poor outcomes. Furthermore, although our patient cohort was highly homogeneous and largely exceeded the required sample size, its overall size remains a limitation, although we performed thorough internal validations using cross validation and bootstrapping. Generalizability of the obtained predictive models was supported by cross validation at two levels, in the entire cohort through LASSO feature selection and by a five-fold cross validation, where models generated in the training folds were tested on unseen validation folds. Remarkably, predictive performance was very similar in the entire cohort and in the half-sized randomly generated training folds, while in the unseen validation folds, AUC only moderately decreased to 0.84. The absence of an external cohort is a limitation of this study because we were unable to exactly determine how well our models would generalize to a different population. Therefore, future studies incorporating external cohorts are planned within our European consortium of institutions dedicated to rectal cancer research (the Horizon Europe project STEPUPIORS—101079217) to further validate the generalizability of our radiomics models in broader patient populations. Additionally, while the computational analysis technique is entirely objective, there is still some residual subjectivity involved in tumor VOI selection for radiomics analysis. The retrospective design of the predictive model is another limitation. Also, the feature selection using LASSO is susceptible to multicollinearity and lacks the statistical test for significance. In our study, we found that LASSO effectively handled multicollinearity, as removing inter-correlated features before the LASSO selection did not impact the results. However, the removal of features with no significant correlation with the outcome yielded favorable results, indicating that through feature preprocessing, we successfully addressed a major limitation in the feature selection process. Other studies have employed similar multi-step approaches to feature selection, involving initial steps like the Wilcoxon rank-sum test, Spearman correlation analysis, followed by LASSO, and multivariate logistic regression analysis [35,40].

## 5. Conclusions

We developed very highly dimensional and moderately dimensional predictive models for neoadjuvant chemoradiotherapy (nCRT) response in rectal carcinoma, using clinical parameters and pretreatment MRI radiomics features. These models outperformed the benchmark model relying on clinicopathological parameters alone. We also showed that using all available image filters and high dimensionality improves predictive performance. The combined clinicopathological and radiomics scores obtained in this study provide a foundation for future broader multimodal predictive approaches that also incorporate genomics [14] and proteomics [12] data. Improved prediction of nCRT response might be clinically significant because it may enable personalized treatment decisions such as delaying surgery or using less aggressive postoperative therapies to minimize toxicity in predicted nCRT responders. For predicted non-responders, more intensive therapies with shorter follow-up periods may reduce recurrence risk.

## Figures and Tables

**Table 1:** Patient, disease, treatment, and outcome characteristics.

Features	N (%)
***Age (years)***	
Mean (SD) (Range)	60.8 (10.6) (33.0–81.0)
***Gender***	
Female/Male	25 (33.3%)/50 (66.7%)
***T in clinical TNM***	
T2	2 (2.7%)
T3	64 (85.3%)
T4	9 (12.0%)
***N in clinical TNM***	
N0	1 (1.3%)
N1a	3 (4.0%)
N1b	18 (24.0%)
N1c	1 (1.3%)
N2a	22 (29.3%)
N2b	30 (40.0%)
***Tumor differentiation***	
well/moderate/poor	39 (52.0%)/30 (40.0%)/6 (8.0%)
***Mucinous histological type***	
Yes/No	13 (17.3)/62 (85.7)
***Absolute basophil count* (10^9^/L)**	
Mean (SD) (Range)	0.1 (0.1) (0.0–1.0)
***Tumor length* (mm)**	
Mean (SD) (Range)	63.2 (18.6) (24–150)
***CRM status***	
Uninvolved/Involved	36 (48.0%)/39 (52.0%)
***Type of CRM involvement***	
By direct tumor spread	20 (26.7%)
By mesorectal TD or metastatic LN	15 (20.0%)
Both categories	4 (5.3%)
Uninvolved	36 (48.0%)
***Extramural vascular invasion (EMVI)***	
Yes/No	
***Surgical treatment***	
No (cCR)/Yes	12 (16.0%)/63 (84.0%)
***TRG score (Surgically treated patients)***	
TRG1	13/63 (20.6%)
TRG2	10/63 (15.9%)
TRG3	30/63 (47.6%)
TRG4	10/63 (15.9%)
***Response to the treatment***	
R (cCR + TRG1 + TRG2)	35/75 (46.7%)
NonR (TRG3 + TRG4)	40/75 (53.3%)

Abbreviations: CRM, circumferential resection margin; TD, tumor deposits; LN, lymph nodes; cCR, patients without operative treatment due to complete clinical response; TRG, tumor regression grade; R, responders; NonR, non-responders.

**Table 2:** Univariate evaluation of the features most strongly associated with chemoradiotherapy response in the three indicated groups of features ^a,b^.

Feature	Pearson ^c^	*p*	R^2^	*p*
**Clinicopathological features**				
Mucinous histological type	0.396	<0.001	0.157	<0.001
N stage	0.302	<0.001	0.091	0.010
Extramural vascular invasion	0.293	<0.001	0.086	0.013
Type of CRM involvement	0.278	<0.001	0.078	0.020
Initial basophil count	0.275	<0.001	0.076	0.020
**Features calculated in original images**				
Original_shape_Max2DDiameterSlice	0.387	<0.001	0.150	<0.001
Original_GLSZM_SizeZoneNonUniformity	0.382	<0.001	0.146	<0.001
Original_GLSZM_SmallHighGrayLevelEmphasis	0.373	<0.001	0.140	<0.001
Original_GLDM_SmallDepHighGrayLevEmphasis	0.367	<0.001	0.135	<0.001
Original_NGTDM_Complexity	0.356	<0.001	0.127	<0.001
**Features calculated in original and filtered images**				
Sqroot_GLDM_SmallDepHighGrayLevEmphasis	0.401	<0.001	0.161	<0.001
Squareroot_GLSZM_SmallHighGrayLevelEmphasis	0.398	<0.001	0.158	<0.001
Exponential_GLSZM_GrayLevelNonUniformity	0.377	<0.001	0.143	<0.001
Logarithm_firstorder_90Percentile	0.375	<0.001	0.141	<0.001
Squareroot_firstorder_90Percentile	0.372	<0.001	0.139	<0.001

^a^ Univariate linear regression test. ^b^ Groups of features are highlighted by gray shading. ^c^ Pearson correlation coefficient. Abbreviations: CRM, circumferential resection margin.

**Table 3:** Predictive models incorporating clinicopathological and radiomics features obtained using LASSO and multivariate linear regression ^a,b,c^.

Feature	Coefficient ^d^ 95% CI	*p* Value
**Model: from 72 CP features** R^2^ = 0.30 ^b^		
Mucinous histological type	Coefficient = 0.478 0.277–0.697	*p* = 0.001
Extramural vascular invasion	Coefficient = 0.432 0.174–0.704	*p* = 0.001
Type of CRM involvement	Coefficient = 0.322 0.058–0.600	*p* = 0.027
**Model: from 72 CP + 107 radiomics features** R^2^ = 0.45		
Mucinous histological type	Coefficient = 0.396 0.163–0.603	*p* = 0.001
Initial basophil count	Coefficient = 0.395 0.249–1.301	*p* = 0.025
N stage	Coefficient = 0.393 0.110–0.653	*p* = 0.010
Original_NGTDM_Complexity	Coefficient = 0.385 0.111–0.581	*p* = 0.002
Original_firstorder_90Percentile	Coefficient = 0.218 −0.006–0.482	*p* = 0.049
**Model: from 72 CP + 1862 radiomics features** R^2^ = 0.47		
Mucinous histological type	Coefficient = 0.258 0.068–0.431	*p* = 0.009
Initial basophil count	Coefficient = 0.384 0.270–1.207	*p* = 0.0014
Type of CRM involvement	Coefficient = 0.319 0.071–0.542	*p* = 0.008
Squareroot_firstorder_90Percentile	Coefficient = 0.483 0.262–0.659	*p* = 0.001
Exponential_GLSZM_GrayLevelNonUniformity	Coefficient = 0.377 0.178–0.528	*p* = 0.001

^a^ Variable selection was performed by the use of the linear LASSO regression, followed by multivariate stepwise linear regression based on probability of *p* = 0.05 for entry and *p* = 0.05 for removal. Univariate predictive evaluation was performed by linear regression. ^b^ Nagelkerke R Square is used in linear regression to assess the goodness-of-fit of the model. ^c^ Models are highlighted by gray shading. ^d^ Due to the z-score normalization of feature values, the coefficients calculated by the multivariate linear regression analysis reflect the relative predictive importance of features. Abbreviations: R^2^, R-square calculated by the univariate linear regression analysis; CRM, circumferential resection margin.

**Table 4:** The predictive performance of the predictive model selected from all CP and radiomics features in the training and validation folds ^a^.

Feature	Average AUC ± SD	*p* Value
Training cohort	0.908 ± 0.010	*p* < 0.000
Validation cohort	0.843 ± 0.020	*p* < 0.001

^a^ A five-fold cross-validation feature selection was performed using LASSO in each of the five produced training folds. The generated models were then tested on the matching and unseen validation folds. Abbreviations: CP, clinicopathological; SD, standard deviation.

## Data Availability

The data presented in this study are openly available in Zenodo, at doi: 10.5281/zenodo.8379940.
